# Supplementary material for: The nematode (Ascaris suum) intestine is a location of synergistic anthelmintic effects of Cry5B and levamisole
Source: bioRxiv. 2023 Nov 20:2023.11.20.567786. Preprint. [Version 1] doi: 10.1101/2023.11.20.567786 (PMC10690214; doi:10.1101/2023.11.20.567786)
Supplement: 1 [file NIHPP2023.11.20.567786V1-supplement-1.pdf]

Supplementary Figure 1

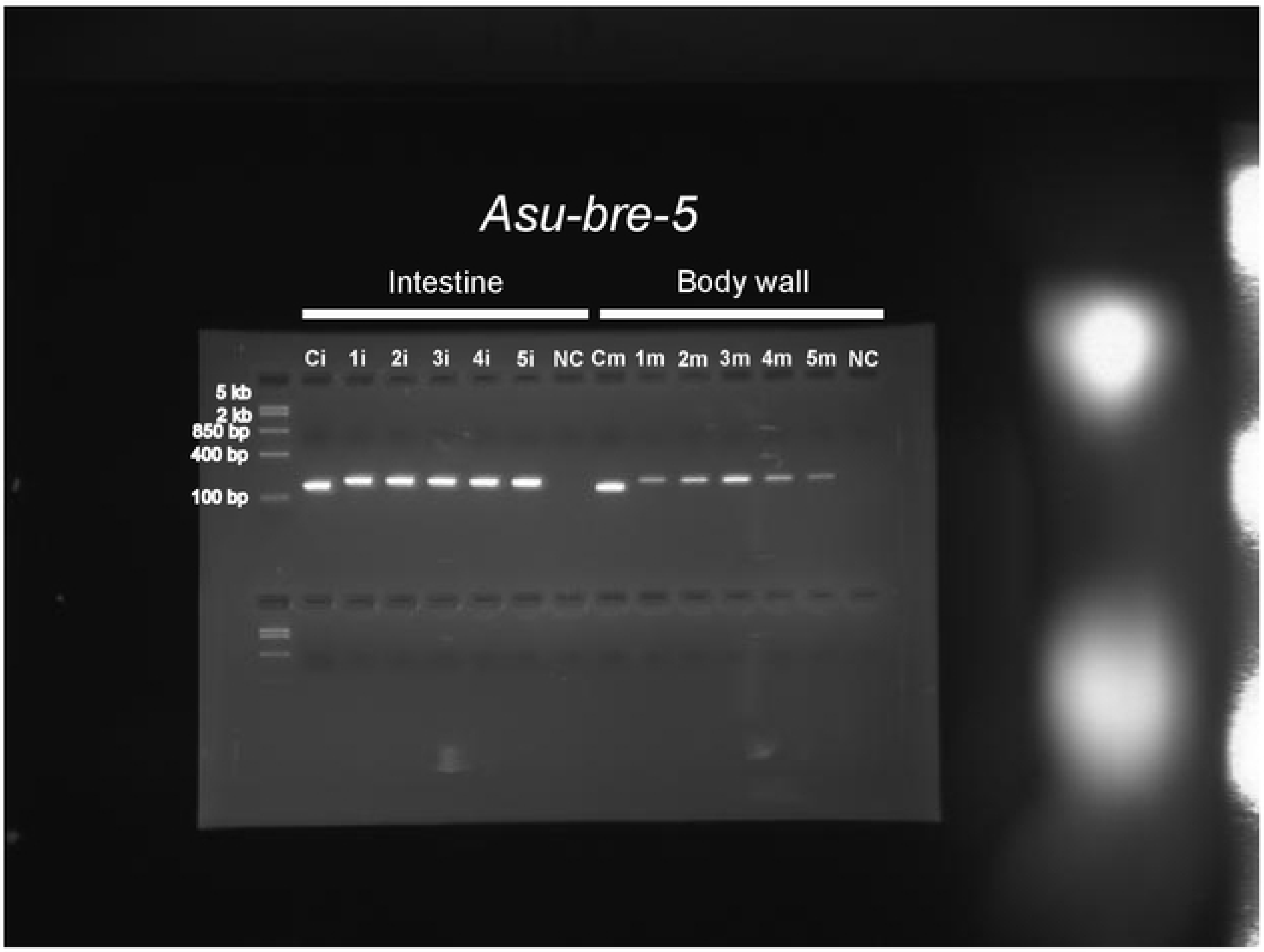

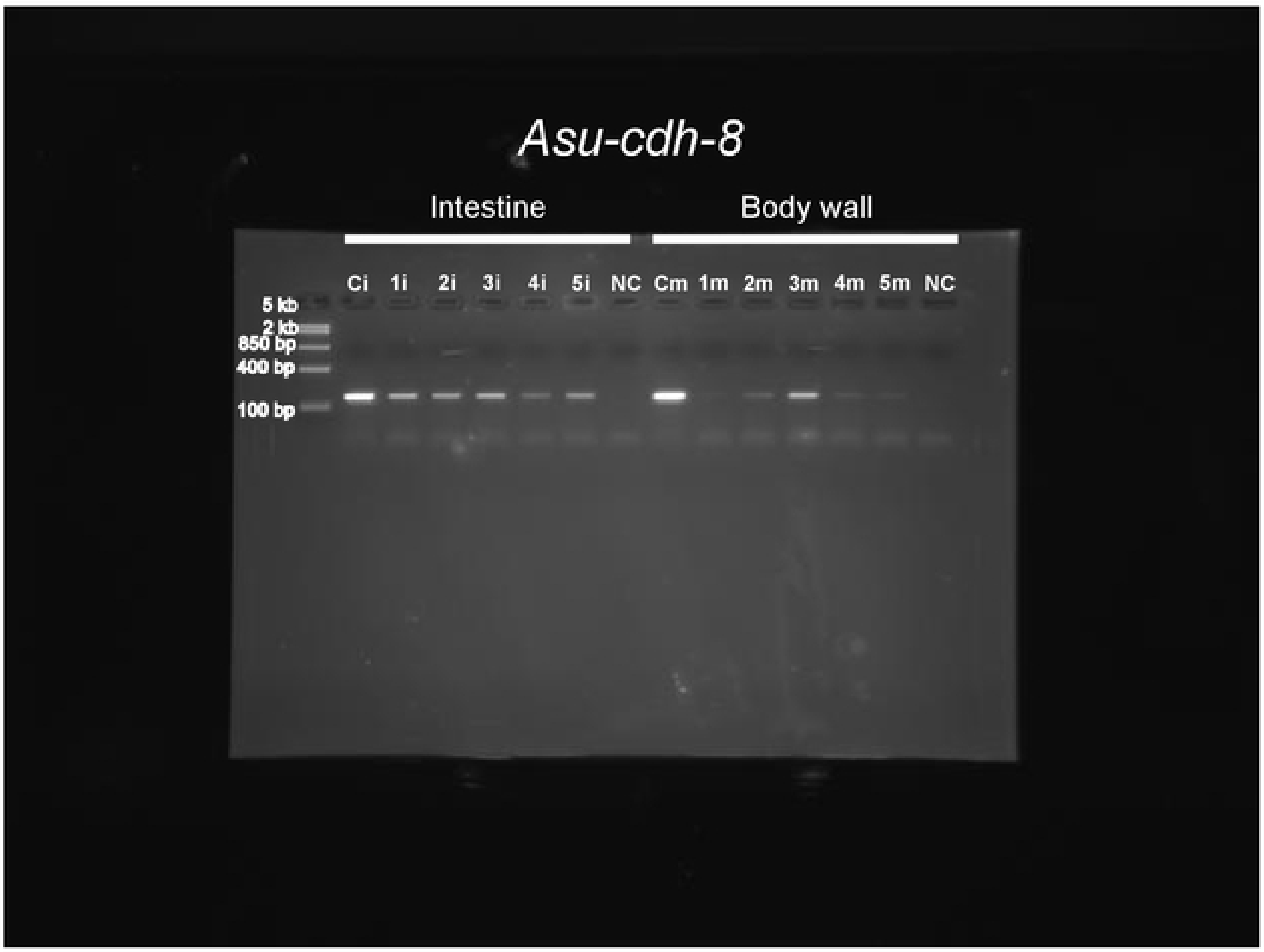

Supplementary Table 1

Gene	Forward Primer	Reverse Primer
Asu-bre-5	AGCACATGGCATAATGTTCC	TTTCTTCGCTCTCAGAAACG
Asu-cdh-8	TCTCAGAGCGGACAAATGTT	AGATGGGATCGTTGTCGTTA
Asu-gapdh	AGCAAGGACCCCTCTGAGAT	TCTCCAGTCTCGCCGTAAGA

Supplementary Table 2

Gene	Forward Primer	Reverse Primer
Asu-bre-5	ATTGAATTTTGGTGGTGTGG	GGCTCGGAAAGTTCAAAAAG
Asu-cdh-8	GTTTACGATCGTTGGAATCG	ATCGGATGCCACAATAAAGA
Asu-gapdh	TCTCGAATGCATCCTGCACC	CACGTCCATCTCTCCATTGC

Supplementary Table 3

Gene	Accession number
Asu-bre-5	AgB01_g494/AEUI03000001.1
Asu-cdh-8	AgR004_g364/AEUI03000009.1
